# Approach to the Patient With Suspected Silver-Russell Syndrome

**DOI:** 10.1210/clinem/dgae423

**Published:** 2024-06-18

**Authors:** Uttara Kurup, David B N Lim, Helena Palau, Avinaash V Maharaj, Miho Ishida, Justin H Davies, Helen L Storr

**Affiliations:** Centre for Endocrinology, William Harvey Research Institute (WHRI), Charterhouse Square, Barts and the London School of Medicine, London EC1M 6BQ, UK; Paediatric Endocrinology, University Hospital Southampton NHS Foundation Trust, Southampton SO16 6YD, UK; Centre for Endocrinology, William Harvey Research Institute (WHRI), Charterhouse Square, Barts and the London School of Medicine, London EC1M 6BQ, UK; Centre for Endocrinology, William Harvey Research Institute (WHRI), Charterhouse Square, Barts and the London School of Medicine, London EC1M 6BQ, UK; Centre for Endocrinology, William Harvey Research Institute (WHRI), Charterhouse Square, Barts and the London School of Medicine, London EC1M 6BQ, UK; Paediatric Endocrinology, University Hospital Southampton NHS Foundation Trust, Southampton SO16 6YD, UK; Faculty of Medicine, University of Southampton, Southampton SO16 6YD, UK; Centre for Endocrinology, William Harvey Research Institute (WHRI), Charterhouse Square, Barts and the London School of Medicine, London EC1M 6BQ, UK

**Keywords:** Silver-Russell syndrome, diagnosis, genetic, NH-CSS

## Abstract

Silver-Russell syndrome (SRS) is a clinical diagnosis requiring the fulfillment of ≥ 4/6 Netchine-Harbison Clinical Scoring System (NH-CSS) criteria. A score of ≥ 4/6 NH-CSS (or ≥ 3/6 with strong clinical suspicion) warrants (epi)genetic confirmation, identifiable in ∼60% patients. The approach to the investigation and diagnosis of SRS is detailed in the only international consensus guidance, published in 2016. In the intervening years, the clinical, biochemical, and (epi)genetic characteristics of SRS have rapidly expanded, largely attributable to advancing molecular genetic techniques and a greater awareness of related disorders. The most common etiologies of SRS remain loss of methylation of chromosome 11p15 (11p15LOM) and maternal uniparental disomy of chromosome 7 (upd(7)mat). Rarer causes of SRS include monogenic pathogenic variants in imprinted (*CDKN1*C and *IGF2)* and non-imprinted (*PLAG1* and *HMGA2*) genes. Although the age-specific NH-CSS can identify more common molecular causes of SRS, its use in identifying monogenic causes is unclear. Preliminary data suggest that NH-CSS is poor at identifying many of these cases. Additionally, there has been increased recognition of conditions with phenotypes overlapping with SRS that may fulfill NH-CSS criteria but have distinct genetic etiologies and disease trajectories. This group of conditions is frequently overlooked and under-investigated, leading to no or delayed diagnosis. Like SRS, these conditions are multisystemic disorders requiring multidisciplinary care and tailored management strategies. Early identification is crucial to improve outcomes and reduce the major burden of the diagnostic odyssey for patients and families. This article aims to enable clinicians to identify key features of rarer causes of SRS and conditions with overlapping phenotypes, show a logical approach to the molecular investigation, and highlight the differences in clinical management strategies.

## Case 1

A 5-year-old girl with a history of intrauterine growth retardation (IUGR) (birth weight −3.8 SDS) had short stature (height −3.9 SDS), feeding difficulties (body mass index [BMI] −3.0 SDS) and microcephaly (occipito-frontal circumference [OFC] −4.9 SDS). Maternal height was reduced (−3.5 SDS) but paternal height was normal (−0.2 SDS). She scored ≥ 3 on the *IGF1R* clinical score (Table 1) (1) but had syndromic features not typical of insulin-like growth factor 1 receptor (IGF1R) defects, including triangular face, high-pitched voice, high-arched palate, and developmental delay (inattention and poor motor, writing, and reading skills). Investigations established normal female karyotype (46,XX) and short stature screen except for elevated serum insulin-like growth factor 1(IGF-1) levels (+4.4 SDS). Silver-Russell syndrome (SRS) was suspected, and she scored 3/6 Netchine-Harbison Clinical Scoring System (NH-CSS) criteria. What would you do next?

**Table 1.** ***IGF1R* clinical scoring system (** 1 **)**

Criterion	Score
Birth weight and/or birth length SDS below −1.0	1
Height SDS at presentation below −2.5	1
Head circumference SDS at presentation below −2.0	1
IGF-1 SDS >0	1
**Total score**	**Score of ≥ 3 should warrant *IGF1R* gene analysis**

## Case 2

A 4-year-old girl with short stature (height SDS −2.9) had a history of birth at 29 weeks’ gestation following IUGR (birth weight and length −1.4 and −2.2 SDS). At birth she had a prominent forehead, relative macrocephaly (head circumference [OFC] + 1.3 SDS) and no body asymmetry. During the neonatal period she was hypotonic and required nasogastric feeding. For the first year following nasogastric tube removal, she was slow to complete feeds. She did not cry during vaccinations, appearing to have a high pain threshold. Height and weight were persistently < 0.4th centile (< −2.7 SDS). Speech and language development was delayed. By 3 years she ate a varied diet and was persistently hungry. At 4 years, her BMI was elevated (+2.5 SDS). Investigations established normal female karyotype (46,XX) and short stature screen. She scored 4/6 on the NH-CSS, confirming a clinical diagnosis of SRS. Molecular testing did not reveal 11p15 hypomethylation or maternal uniparental disomy of chromosome 7. Recombinant growth hormone (rhGH) was commenced under the small for gestational age (SGA) license. What other diagnostic molecular testing should be considered at the same time?

## Short Stature Secondary to Being Born Small for Gestational Age—Definition and Clinical Relevance

When assessing childhood short stature, a key consideration is a history of IUGR and/or born SGA (defined as a birth weight and/or length < −2 SDS). Children with short stature, secondary to being born SGA, comprise an extremely heterogeneous group with numerous underlying causes of both syndromic and nonsyndromic phenotypes. SRS and conditions that mimic SRS are increasingly recognized as an important cause of short stature secondary to being born SGA. It is critical to distinguish SRS from these other conditions. (Epi)genetic investigations can be key to stratification of these disorders and, in turn, a more tailored clinical management.

## Silver-Russell Syndrome

SRS is characterized by pre- and postnatal growth restriction, dysmorphic features, and feeding difficulties. SRS occurs equally in male and female individuals and has an estimated incidence of 1 in 15 000 children (2). SRS can usually be distinguished from other causes of pre- and postnatal growth failure by the presence of SGA, relative macrocephaly (head circumference at birth of ≥ 1.5 SDS above birth and/or length SDS), prominent forehead, feeding difficulties, and body asymmetry.

SRS has a diverse phenotype (Tables 2 and 3), and some clinical characteristics are less apparent with increasing age, thus making a clinical diagnosis challenging in older people (31). The age-specific NH-CSS has the highest sensitivity and predictive value for making a clinical diagnosis of SRS (Table 2). Essential features of the NH-CSS are SGA at birth, postnatal growth failure by 2 years, asymmetry, feeding difficulties in the first 2 years of life, prominent forehead between 1 and 3 years, and relative macrocephaly at birth. A clinical diagnosis of SRS requires the fulfillment of ≥ 4/6 NH-CSS criteria, including relative macrocephaly and prominent forehead. (Epi)genetic testing is recommended for patients who score ≥ 4/6 (or ≥ 3/6 with strong clinical suspicion) (2).

**Table 2.** NH-CSS criteria for clinical Silver-Russell syndrome (SRS) diagnosis

Diagnostic features of SRS (NH-CSS criteria)	Clinical features associated with SRS but not specific to SRS
Small for gestational age (birth weight and/or length ≥ 2 SDS below the mean for gestational age)	Triangular face Fifth finger clinodactyly Shoulder dimples Micrognathia Low muscle mass Excessive sweating Low-set and/or posteriorly rotated ears Downturned mouth High-pitched or squeaky voice Prominent heels Delayed closure of fontanelle Male genital abnormalities Speech delay Irregular or crowded teeth Motor delay Syndactyly of toes Hypoglycemia Scoliosis and/or kyphosis
Postnatal growth failure (length/height ≥ 2 SDS below the mean at 24 months)	
*^a^* Relative macrocephaly at birth (head circumference > 1.5 SDS above birth weight and/or length)	
*^a^* Frontal bossing or prominent forehead (forehead projecting beyond the facial plane on a side view as a toddler [1-3 years])	
Body asymmetry (limb length discrepancy ≥ 0.5 cm, or < 0.5 cm with ≥ 2 other asymmetric body parts)	
Feeding difficulties or body mass index ≤ 2 SD at 24 months or current use of a feeding tube or cyproheptadine for appetite stimulation	

^*a*^Major criteria for the clinical SRS diagnosis. SRS should be suspected in individuals who meet ≥ 4/6 clinical criteria. Those who meet at least 3 of the clinical criteria with a high suspicion need molecular testing to confirm the diagnosis (3).

**Table 3.** Comparison of clinical features in Silver-Russell syndrome patients with confirmed 11p15LOM, mUPD7, and monogenic causes

Clinical feature	(Epi)genetic cause of Silver-Russell syndrome					
	11p15LOM	upd(7)mat	*CDKN1C*	*IGF2*	*HMGA2*	*PLAG1*
**NH-CSS % (n)**						
Patients scoring ≥ 4/6	78% (61)	85% (17)	53% (9)	86% (18)	65% (11)	40% (4)
Patients scoring 3/6	22% (17)	15% (3)	47% (8)	9% (2)	23% (4)	50% (5)
Patients scoring < 3/6	NR	NR	NR	5% (1)	12% (2)	10% (1)
SGA (birth weight and / or birth length)*^a^*	100% (35)	73% (11)	71% (12)	95% (20)	88% (15)	80% (8)
Postnatal growth failure*^b^*	84% (173)	81% (47)	100% (17)	95% (20)	100% (17)	100% (10)
Relative macrocephaly at birth*^c^*	99% (112)	85% (27)	59% (10)	81% (17)	29% (5)	20% (2)
Protruding/prominent forehead*^d^*	94% (126)	100% (27)	94% (16)	86% (18)	71% (12)	60% (6)
Body asymmetry*^e^*	77% (226)	29% (62)	NR	29% (6)	6% (1)	NR
Feeding difficulties and / or low BMI*^f^*	72% (173)	87% (47)	59% (10)	86% (18)	71% (12)	90% (9)
**SRS associated features % (n)**						
Triangular face	99% (74)	50% (16)	76% (13)	86% (18)	65% (11)	70% (7)
Delayed closure of fontanelle	44% (36)	36% (11)	6% (1)	5% (1)	NR	NR
Low-set +/− posteriorly rotated ears	50% (140)	69% (48)	6% (1)	38% (8)	NR	NR
Downturned mouth	57% (114)	26% (39)	NR	9% (2)	NR	NR
Irregular/crowded teeth	29% (105)	39% (36)	6% (1)	5% (1)	6% (1)	20% (2)
Micrognathia	75% (79)	26% (27)	NR	48% (10)	6% (1)	10% (1)
Low muscle mass	67% (61)	47% (19)	NR	9% (2)	6% (1)	NR
Fifth finger clinodactyly	81% (176)	56% (48)	6% (1)	71% (15)	24% (4)	30% (3)
Syndactyly of toes	42% (141)	17% (48)	NR	19% (4)	6% (1)	NR
Prominent heels	26% (35)	100% (12)	NR	NR	NR	NR
Shoulder dimples	77% (35)	67% (12)	NR	5% (1)	NR	NR
Scoliosis +/− kyphosis	10% (97)	16% (43)	NR	NR	NR	NR
High-pitched / squeaky voice	39% (26)	71% (7)	NR	24% (5)	12% (2)	NR
Excessive sweating	51% (70)	70% (27)	NR	NR	NR	NR
Hypoglycemia	50% (43)	29% (17)	NR	NR	NR	NR
**Other features % (n)**						
Motor delay	31% (141)	58% (36)	6% (1)	62% (13)	6% (1)	30% (3)
Speech delay	32% (101)	64% (36)	NR	52% (11)	NR	30% (3)
Intellectual disability / Learning difficulties	NR	NR	NR	5% (1)	NR	10% (1)
Challenging behavior/inattention	6% (35)	58% (12)	6% (1)	NR	NR	NR
Gastrointestinal manifestations / gastroesophageal reflux	14% (6)	10% (2)	NR	NR	12% (2)	10% (1)
Diabetes	NR	NR	12% (2)	NR	6% (1)	10% (1)
Delayed bone age	NR	NR	NR	24% (5)	18% (3)	NR
Asthma	NR	NR	12% (2)	NR	NR	NR
Cardiac abnormalities	13% (7)	NR	NR	43% (9)	NR	NR
Cleft palate	7% (3)	NR	NR	29% (6)	NR	NR
Male genital abnormalities	44% (63)	21% (14)	6% (1)	33% (7)	NR	NR
Placental hypoplasia / small placenta	NR	NR	NR	17% (5)	NR	10% (1)
References	(4-11)	(4-12)	(13-18)	(16, 17, 19-21)	(19, 22-27)	(17, 19, 28-30)

Literature review of clinical features of SRS associated with confirmed 11p15LOM, mUPD7, and monogenic causes. N (%), number (and percentage of total) cases exhibiting the feature. Duplicate reporting of cases was not identified.

Abbreviations: 11p15LOM, loss of methylation of chromosome 11p15; CDK1N1C, Cyclin-dependent kinase inhibitor 1; HMGA2, high-mobility group AT-hook 2; IGF2, insulin-like growth factor 2; NH-CSS, Netchine-Harbison Clinical Scoring System; NR, not recorded; PLAG1, pleomorphic adenoma gene 1; SGA, small for gestational age; SRS, Silver-Russell syndrome; upd(7)mat, maternal uniparental disomy of chromosome 7.

NH-CSS criteria: *^a^*SGA, defined as birth weight/length ≤ −2 SDS for gestational age; *^b^*Postnatal growth failure, defined as height ≤ −2 SDS or height ≤ −2 SDS below mid-parental target height at 24 ± 1 months; *^c^*Relative macrocephaly at birth, defined as head circumference at birth ≥1.5 SDS above birth weight and/or length SDS; *^d^*Prominent forehead, defined as forehead projecting beyond the facial plane on a side view as a toddler (1-3 years); *^e^*Asymmetry, defined as leg length discrepancy (LLD) of ≥ 0.5 cm or arm asymmetry or LLD < 0.5 cm with at least 2 other asymmetrical body parts (one non-face); ^f^Low BMI, defined as body mass index ≤−2 SDS at 24 months or use of a feeding tube or cyproheptadine for appetite stimulation; microcephaly, occipito-frontal circumference (OFC) > 2 SD below the mean for age, sex, and ethnicity.

## Etiology of SRS

A diagnosis of SRS can be based on an identifiable molecular defect (∼30%-60% of cases) or a clinical diagnosis using standard clinical criteria. Therefore, inconclusive genetic testing does not exclude SRS and occurs in a significant proportion (∼40%) of patients, classified as “clinical SRS” (32) (see later).

**Figure 1.**
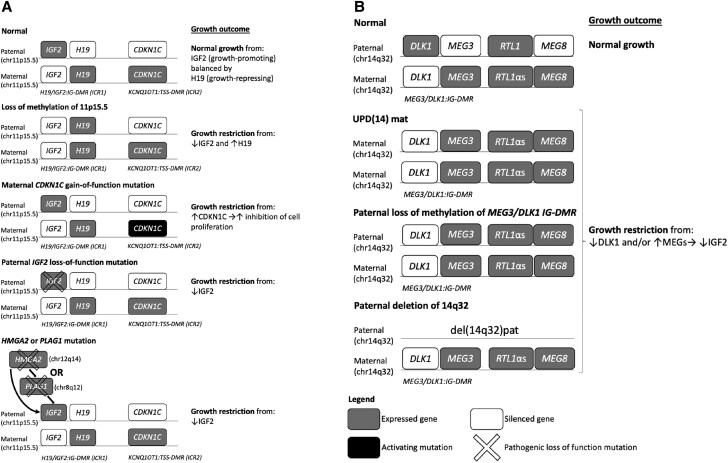
Proposed mechanisms in Silver-Russell syndrome (SRS) and Temple syndrome leading to growth restriction. A, Representation of the 11p15 region, showing the paternal and maternal telomeric H19/IGF2 IG-DMR (ICR1) and centromeric KCNQ1OT1 TSS-DMR (ICR2) imprinting control regions associated with SRS. A differentially paternally methylated region between the IGF2 and H19 genes regulates expression. Aberrant expression of genes (upstream or within the imprinting control region) controlling the IGF2 gene (paternally expressed fetal growth factor) and H19 (maternally expressed) expression leads to growth restriction. B, Representation of the 14q32 region implicated in Temple syndrome (TS14). TS14 can be caused by 3 mechanisms: maternal UPD leading to expression of only maternally expressed genes from both chromosomes, paternal loss of methylation of MEG3/DLK1 IG-DMR resulting in a maternal chromosome-like expression pattern, or deletion in the paternal chromosome resulting in absence of paternally expressed genes. Aberrant expression of these genes is associated with IGF2 downregulation, leading to growth restriction. Gray boxes indicate expressed genes; open boxes, silenced genes; black box, activating mutation (CDKN1C); X, pathogenic loss-of-function mutations (HMGA2, IGF2, PLAG1).

In ∼60% of clinically diagnosed SRS patients, an (epi)genetic cause is identified. SRS is molecularly heterogenous; the most common causes are loss of methylation of chromosome 11p15 (11p15LOM) (Fig. 1A) and maternal uniparental disomy of chromosome 7 (upd(7)mat), occurring in 30% to 60% and 5% to 10% of SRS cases, respectively. 11p15LOM results in reduced paternal *IGF2* and increased maternal *H19* expression, leading to growth restriction (Fig. 1A) (33). Rarer genetic causes of SRS include monogenic pathogenic variants in imprinted (*CDKN1*C and *IGF2)* and non-imprinted (*PLAG1* and *HMGA2*) genes and copy number variants (CNV) (34, 35). Growth restriction observed in SRS due to monogenic variants result from: inhibition of cell proliferation due to maternally inherited gain-of-function *CDKN1*C defects (13), or reduction in *IGF2* levels secondary to paternally inherited loss-of-function *IGF2* defects, and pathogenic *PLAG1* / *HMGA2* gene variants (19) (Fig. 1A).

### Common Molecular Causes of SRS

The frequency of associated clinical features in SRS subgroups (11p15LOM, upd(7)mat, and clinical SRS) and patients with SGA but not SRS, was extensively reviewed in the SRS consensus document (1). Genotype-phenotype studies indicate considerable overlap in the clinical phenotypes of SRS (epi)genotypes and are generally considered clinically indistinguishable. However, some features are more common in the molecular subgroups, and these are highlighted below.

#### Loss of methylation of chromosome 11p15

NH-CSS criteria are generally better at detecting SRS secondary to 11p15LOM compared to upd(7)mat (4, 5). Individuals exhibiting 11p15LOM typically have lower birth length and weight and a higher incidence of body asymmetry and congenital anomalies than upd(7)mat patients. Excessive catch-up weight gain leading to increased metabolic and cardiovascular risk is more frequent in 11p15LOM compared to upd(7)mat. Genital ambiguity is more frequent in patients with severe 11p15LOM and includes females with uterine and vaginal aplasia and cryptorchidism and hypospadias in male patients (5, 36, 37). Serum IGF-1 levels are significantly higher in 11p15LOM epimutation patients than in those with upd(7)mat (38).

Two imprinted domains on chromosome 11p15.5 play a crucial role in regulating intrauterine growth (32, 39) (Fig. 1A). The telomeric domain implicated in SRS is regulated by the paternally methylated imprinting control region H19/IGF2 IG-DMR (H19/IGF2 intergenic differentially methylated region). Hypomethylation of the H19/IGF2 IG-DMR results in reduced paternal Insulin-like Growth Factor 2 (*IGF2*) expression and increased maternal noncoding RNA H19 expression, leading to growth repression (40). Hypomethylation typically affects both IGF2 and H19, rarely affecting one of them exclusively. The likelihood of SRS recurrence in siblings or offspring of probands is low, except when there are CNVs in this region (32). Multiple DNA methylation abnormalities, collectively termed multi-locus imprinting disturbance (MLID), has been documented in up to 30% of individuals diagnosed with SRS (see below).

#### Maternal uniparental disomy of chromosome 7

Neurocognitive impairment is more prevalent among individuals with upd(7)mat in contrast to either 11p15LOM or clinical SRS patients. Furthermore, upd(7)mat is also associated with pervasive developmental disorders such as autism and learning disabilities (2).

The SRS phenotype associated with upd(7)mat is hypothesized to result from the altered expression of currently unknown growth gene(s). Pathogenic gene mutations were excluded in candidate regions 7p11.2-p13 (*GRB10*) and 7q31 (*MEST/PEG1*) (41). Overexpression of maternal growth limiting genes (*MEST/PEG1*) may be the primary cause of maternal uniparental disomy at chromosome 7q32 rather than reduced expression or deletion of paternal growth promoting gene(s) (40). The imprinting control region 7p12.1 (*GRB10*) has also been implicated in upd(7)mat. Hypomethylation can occur due to maternal isodisomy/heterodisomy, mosaicism, or segmental upd(7)mat. Isodisomy can lead to the expression of recessive conditions such as cystic fibrosis in upd(7)mat SRS (42). The risk of recurrence in siblings or offspring of probands is low, except in cases of microdeletions or microduplications (up to ∼50% recurrence based on parental carrier or affected status) (32).

### Rarer (Monogenic) Causes of SRS

Monogenic causes of SRS can be challenging to diagnose since features not typically associated with SRS may be present and the clinical presentation may not fulfill the NH-CSS criteria. Molecular testing is therefore recommended in patients scoring ≥ 3/6 NH-CSS (2). Monogenic causes of SRS are rare and exhibit autosomal dominant inheritance (14). SRS due to monogenic defects have been identified in Cyclin-dependent kinase inhibitor 1C (maternally transmitted; *CDKN1C*), insulin-like growth factor 2 (paternally transmitted; *IGF2)*, pleomorphic adenoma gene 1 (*PLAG1*), and high-mobility group AT-hook 3 (*HMGA2*) (Fig. 1A). Unlike *IGF2* and *CDKN1*C, *HMGA2* and *PLAG1* are not imprinted and both male and female individuals have a 50% chance of transmitting the mutation (19, 28, 43).

#### Cyclin-dependent kinase inhibitor 1

Review of 17 reported *CDKN1C* gene defect cases with detailed clinical data revealed 47% did not fulfill the NH-CSS criteria (scoring 3/6) (Supplementary Table S1 (44)). Relative macrocephaly and prominent forehead were noted in 59% and 94% of cases, respectively. No cases had microcephaly (defined as OFC ≥ 2 SD below the mean for age, sex, and ethnicity). Postnatal growth failure was present in all cases and 71% of patients were born SGA. Feeding difficulties were frequent (59%) but none presented with body asymmetry. Other clinical features not typically associated with SRS included diabetes (12%), motor and/or speech delay (6%), challenging behavior and poor concentration (6%), and asthma (12%) (Table 3 and Supplementary Table S1) (13-18, 44, 45).

*CDKN1C* is a maternally expressed imprinted growth repressor gene located in the centromeric domain of chromosome 11p15) (Fig. 1A). *CDKN1C* imprinting is controlled by the imprinting control region KCNQ1OT1 TSS-DMR. *CDKN1C* is involved in cell proliferation inhibition through G1 cell cycle arrest. Gain-of-function mutations in this region lead to growth restriction observed in SRS and IMAGe Syndrome (13, 46). In contrast, loss-of-function *CDKN1C* mutations are associated with an overgrowth phenotype of Beckwith-Wiedemann syndrome. Monoallelic dominant mutations lead to increased stability of the CDKN1C protein and functional gain (15).

Gain-of-function mutations affecting the highly conserved 279th amino acid within analogous proliferating cell nuclear antigen (PCNA) domains of the *CDKN1C* gene have been reported in IMAGe syndrome and familial SRS (p.Arg279Pro, p.Arg279Ser, p.Arg279Leu) (15). *CDKN1C* c.843G>T (p.Arg281Leu) cases are also associated with early-onset adult diabetes (18).

#### Insulin-like growth factor 2

Of 21 patients harboring pathogenic *IGF2* variants, 86% presented with high NH-CSS scores (≥ 4/6) (Supplementary Table S2 (44)). Most patients were born SGA (95%), had postnatal growth failure (95%), relative macrocephaly (81%), and prominent forehead (86%). There was also a high frequency of feeding issues (86%). There were no cases of microcephaly and body asymmetry was infrequent (29%). Interestingly, there was a high percentage of clinical features not typically associated with SRS, including developmental delay (speech/motor) (62%), male genital abnormalities (33%), cardiac anomalies (43%), cleft palate (29%), and intellectual disabilities (5%) (Table 3 and Supplementary Table S2) (16, 17, 19-21, 29, 44, 47-52). Biochemical analyses revealed higher serum IGF-1/IGFBP3, lower IGF2 levels, and a lower IGF2/IGF-1 ratio (47, 53).

*IGF2,* located on chromosome 11p15, is maternally imprinted (paternally transmitted) and biparentally expressed in the liver and brain (Fig. 1A). Unlike 11p15 LOM, *IGF2* defects do not exhibit somatic mosaicism (47), which may explain the phenotypic differences described above. The reported SRS-causing *IGF2* gene mutations are splice site (leading to exon skipping), nonsense or frameshift (leading to nonsense mediated mRNA decay) mutations (17). IGF2 binds to the insulin-like growth factor binding protein (IGFBP), preventing spontaneous degradation. *IGF2* mutations that influence this essential binding promote instability and thus increased *IGF2* clearance/reduced half-life, resulting in diminished placental and fetal cell growth. This is evidenced by placental hypoplasia (21, 47).

#### High-mobility group AT-hook 2

Review of 17 reported cases of SRS secondary to *HMGA2* defects revealed that 35% scored ≥3/6 NH-CSS (Supplementary Table S3 (44)). Prominent forehead was a frequent feature (71%) but only 29% had relative macrocephaly. Interestingly, microcephaly was present in 29%. All cases had postnatal growth failure, 88% associated with being born SGA. Feeding difficulties were reported in 71% patients but body asymmetry was infrequent (6%) (Table 3 and Supplementary Table S3) (19, 22-27, 44).

*HMGA2* gene expression reduces with advancing age, with highest expression identified in fetal tissues (22). HMGA2 and PLAG1 play independent and cumulative roles in the regulation of IGF2 in the HMGA2-PLAG1-IGF2 pathway (19). *HMGA2* silencing initiates a reduction in *PLAG1* and consequently a reduction in total IGF2 levels (Fig. 1A). PLAG1 binds to the IGF2 promoter P3, which is highly expressed in fetal tissue. Lower *IGF2* promoter P3 levels are associated with *HMGA2* silencing when compared to *PLAG1* mutations, highlighting its independent role in *IGF2* transcription. Heterozygous missense and nonsense mutations both within and outside the AT-hooks have been reported in SRS. The severity of phenotypic abnormalities is potentially determined by the number of residual functional AT-hooks (22).

#### Pleomorphic adenoma gene 1

Review of 10 SRS cases harboring *PLAG1* gene mutations confirmed that 60% scored ≥ 3/6 NH-CSS (Supplementary Table S4 (44)). All had postnatal growth failure, associated with SGA at birth in 80% patients. Prominent forehead was present in 60%, but only 20% of patients had relative macrocephaly. Microcephaly was present in 30% of cases. No patient had body asymmetry, but feeding difficulties were common (90%). Other clinical features not typically associated with SRS included speech and/or motor delay (40%) and gastrointestinal manifestations (10%) (Table 3 and Supplementary Table S4) (17, 19, 28-30, 44).

*Plag1^−/−^* mice showed a reduction in overall weight at birth, continuing into adulthood, compared to *Plag1^+/+^* mice. A similar phenotype was seen in paternal *Igf2*-deficient mice (43). Frameshift and nonsense *PLAG1* mutations affect its DNA-binding ability and lead to reduced levels of IGF2 (Fig. 1A).

#### SRS secondary to copy number variations

CNVs account for ∼1% of SRS cases and more than 30 different pathogenic CNVs have been associated with SRS. Some of these patients do fulfill the NH-CSS criteria and receive a clinical SRS diagnosis. These cases generally have more severe developmental delay and/or intellectual disability than typically observed in SRS; however, the phenotype is dependent on CNV size, location, and parental origin (2, 54). Thus, the clinical management should be tailored to the specific phenotypic consequences of the CNV. The majority of the CNVs involve maternal duplications of H19/IGF2 IG-DMR and/or KCNQ1OT1 TSS-DMR (40, 55).

## Clinical SRS

**Figure 2.**
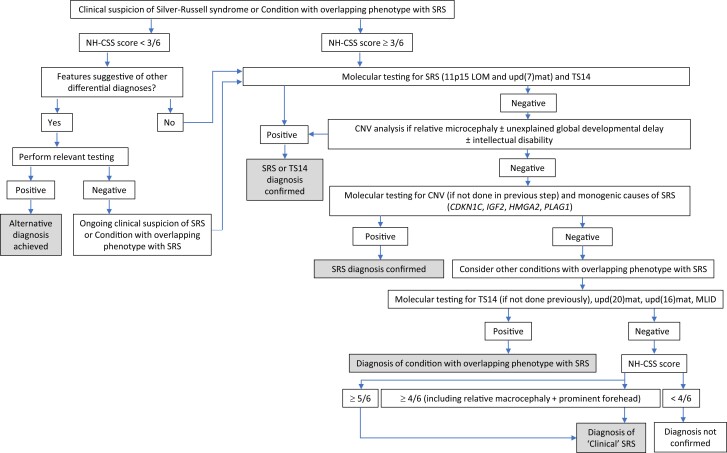
Flow chart for the investigation and diagnosis of Silver-Russell syndrome (SRS) and conditions with phenotypes overlapping with SRS.

A “clinical SRS” diagnosis can be made based on clinical criteria using the NH-CSS, as the identification of a molecular defect is not required to make an SRS diagnosis. In approximately 40% of “clinical” SRS cases fulfilling the NH-CSS criteria, no molecular cause is identified, and in these cases, it is likely that there are currently undiscovered (epi)genetic causes. In clinical SRS cases, there is also the potential for a misdiagnosis of SRS when in fact it may be another distinct condition that has an overlapping phenotype with SRS (Fig. 2).

## Conditions With Phenotypes Overlapping With SRS

Several conditions have clinical features that “mimic” SRS and may fulfill the NH-CSS criteria but that have a genetic etiology and clinical course distinct from SRS. These conditions may be presumptively diagnosed as clinical SRS if standard (epi)genetic investigations for SRS are negative. They are under-recognized, overlooked, and under-investigated by clinicians managing short stature and can lead to delayed or no diagnosis and poorer outcomes.

Evidence is accumulating that these conditions require tailored management and alternative care strategies to SRS. This emphasizes the need to extend the molecular investigation of apparent clinical SRS to inform clinical management decisions. Critically, like SRS, these conditions are multisystemic disorders requiring multidisciplinary care. Table 4 provides a summary of the conditions which have phenotypes overlapping with SRS (2, 56, 57).

**Table 4.** **Conditions with phenotypes overlapping with SRS (** 2, 56, 57**)**

Condition	Molecular mechanism	Possible clinical features overlapping with SRS
*Conditions associated with normocephaly or macrocephaly*		
Temple syndrome	Disruption to 14q32 region of chromosome 14 due to: maternal uniparental disomy of chromosome 14 (30%-78% of cases), paternal hypomethylation of the MEG3/DLK1 IG-DMR (12%-60% of cases), or (iii) paternal deletion of 14q32 (∼10%)	IUGR and SGA Postnatal growth failure Short stature Relative macrocephaly Prominent forehead Asymmetry Feeding difficulties in infancy
UPD(20)mat	Maternal uniparental disomy of chromosome 20	IUGR and SGA Postnatal growth failure Short stature Relative macrocephaly Prominent forehead Feeding difficulties in infancy
UPD(16)mat	Maternal uniparental disomy of chromosome 16	IUGR and SGA Postnatal growth failure Short stature Relative macrocephaly Prominent forehead Feeding difficulties in infancy
MLID	Variable	variable
SHORT syndrome	Heterozygous *PIK3R1* gene mutations (chromosome 5q13)	IUGR and SGA Postnatal growth failure Short stature Triangular face Prominent forehead
Floating-Harbor syndrome	Heterozygous *SRCAP* gene mutations (chromosome 16p11)	IUGR and SGA Postnatal growth failure Short stature Normocephaly Triangular face Fifth finger clinodactyly Brachydactyly Low-set ears Speech and language delay Male genital abnormalities (eg, hypospadias, crypto-orchidism)
3-M syndrome	Homozygous *CCDC8* gene mutations (chromosome 19q13) in 75% of cases; or Homozygous or compound heterozygous *OBSL1* gene mutations (chromosome 2q35) in 20% of cases; or Homozygous *CCDC8* gene mutations (chromosome 19q13) in 5% of cases	IUGR and SGA Postnatal growth failure Short stature Relative macrocephaly Prominent forehead Triangular face Fifth finger clinodactyly Prominent heels
Mulibrey nanism	Homozygous or compound *TRIM37* gene heterozygous mutations (chromosome 17q22)	IUGR and SGA Postnatal growth failure Short stature Relative macrocephaly Prominent forehead Triangular face High-pitched voice
IMAGe syndrome	Heterozygous *CDKN1C* gene mutations (chromosome 11p15)	IUGR and SGA Postnatal growth failure Short stature Male genital abnormalities (eg, micropenis, hypospadias, crypto-orchidism)
*Conditions associated with microcephaly*		
*IGF1* gene defects	Homozygous or heterozygous mutation in *IGF1* gene on chromosome 12q22	IUGR and SGA Postnatal growth failure Short stature Developmental delay
*IGF1R* gene defects	Heterozygous mutation in *IGF1R* gene on chromosome 15q26	IUGR and SGA Postnatal growth failure Short stature Developmental delay
Bloom syndrome	Homozygous or compound heterozygous *RECQL3* gene mutations (chromosome 15q26)	IUGR and SGA Postnatal growth failure Short stature Developmental delay Triangular face Micrognathia Low-set ears Feeding difficulties in infancy
Nijmegen breakage syndrome	Homozygous or compound heterozygous *RAD50* gene mutations (chromosome 5q31)	IUGR and SGA Postnatal growth failure (mild) Short stature Developmental delay (mild) Fifth finger clinodactyly Syndactyly of toes
MOPD II	Homozygous or compound heterozygous *PCNT* gene mutations (chromosome 21q22)	IUGR and SGA Postnatal growth failure (mild) Short stature High-pitched voice Micrognathia Fifth finger clinodactyly Scoliosis
Meier-Gorlin syndrome	Homozygous or compound heterozygous *ORC1* gene mutations (chromosome 1p32); or Homozygous or compound heterozygous *ORC4* gene mutations (chromosome 2q23); or Homozygous or compound heterozygous *ORC6* gene mutations (chromosome 16q11); or Homozygous or compound heterozygous *CDT1* gene mutations (chromosome 16q24); or Homozygous *CDC6* gene mutations (chromosome 17q21); or Homozygous or compound heterozygous *CDC45* gene mutations (chromosome 22q11); or Compound heterozygous *MCM5* gene mutations (chromosome 22q12)	IUGR and SGA Postnatal growth failure Short stature Triangular face Low-set ears Micrognathia Fifth finger clinodactyly Delayed motor and/or speech development Feeding difficulties in infancy Male genital abnormalities (eg, micropenis, hypospadias, crypto-orchidism) Scoliosis

Abbreviations: IUGR, intrauterine growth restriction; SGA, small for gestational age; SRS, Silver-Russell syndrome.

### Temple Syndrome

Temple syndrome (TS14) is caused by disruption to the 14q32 region of chromosome 14 due to: (i) maternal uniparental disomy of chromosome 14 (upd(14)mat) (30%-78% cases); (ii) paternal hypomethylation of the MEG3/DLK1 IG-DMR (12%-60% cases); or (iii) paternal deletion of 14q32 (∼10%) **(**Fig. 1B) (58-60).

The presenting phenotype of TS14 overlaps with SRS, Prader-Willi syndrome (PWS), and SGA-related short stature. Most patients with TS14 presenting in infancy have features of both SRS and PWS (50% cases). Approximately, 20% of cases have an SRS phenotype and may initially be labeled as clinical SRS, while 20% have a PWS phenotype and 10% a short stature SGA only phenotype (59, 61). The overlapping clinical features between these presenting phenotypes make clinical diagnosis challenging and features may become less prominent with increasing age. Body composition changes in children with TS14 (high fat mass and low lean mass) contrasts with that observed in SRS (low fat mass and low lean mass) and may help differentiate TS14 from SRS (61). If genetic testing is negative for infants with suspected SRS or PWS, investigation for TS14 should be considered.

Characteristic clinical features of TS14 include SGA (84%), postnatal growth failure and short stature (92%), relative macrocephaly (52%), prominent forehead (63%) at birth, asymmetry (23%), and feeding difficulties (63%) (59). Thus, some TS14 cases fulfill the NH-CSS. Other features include hypotonia (68%), developmental delay (19%), early-onset obesity and hyperphagia (11%), gonadotrophin-dependent precocious puberty (GDPP), high pain threshold, small hands and/or feet, and psycho-behavioral issues (59, 61).

Feeding difficulties in infancy are frequent and may require nasogastric tube placement, but usually less severe than observed in SRS (58, 61). In older children with TS14, reduced appetite is less problematic to manage compared to SRS. Absence of satiety may manifest from early childhood, but the hyperphagia is usually not as severe as PWS. The nutritional goals change with increasing age and calorie restriction to prevent excess weight gain may be required at an early age to reduce the likelihood of early-onset obesity. Like SRS, surveillance for ketotic hypoglycemia, scoliosis, and metabolic syndrome is needed (62).

GDPP is more prevalent in TS14 (86% of cases) compared to SRS, requiring monitoring from approximately 4 years of age (58, 60, 61). Unlike SRS, premature adrenarche is not a typical feature of TS14. GDPP is amenable to treatment with gonadotropin-releasing hormone analogue (GnRHa) therapy (63). The loss of function of DLK1, located within the 14q32.2 imprinting region, and which is involved in osteogenesis, adipogenesis, and regulation of hypothalamic satiety, is likely a contributory factor to GDPP and obesity (64-66).

Short stature is a frequent feature of TS14. Apparent growth hormone (GH) deficiency demonstrated through failed GH provocation testing, has been observed in up to 15% (n = 2/13) of TS14 cases (59). Some children who have TS14 without GH deficiency may be eligible for rhGH therapy under the SGA indication. Treatment with rhGH improves short-term linear growth and may optimize final height, although, data is limited (67, 68). Combined treatment with rhGH and GnRHa may further improve growth outcomes (59). Growth hormone treatment may also be beneficial for improving body composition.

TS14 secondary to a paternal deletion of 14q32 has been reported in association with thyroid dysfunction and papillary carcinoma at a young age (69). The deleted region in TS14 contains MEG3 (which is thought to result in tumor suppression) and may be implicated in the development of thyroid carcinoma (70). This genotype may require clinical, biochemical, and sonographic monitoring for thyroid disease.

### Maternal Uniparental Disomy of Chromosome 20

Maternal uniparental disomy of chromosome 20 (upd(20)mat or Mulchandani-Bhoj-Conlin syndrome), may account for up to 5% of cases presenting with an SRS phenotype (71). Upd(20)mat has recently been recognized as an imprinting disorder with overlapping features of SRS such as being born SGA, postnatal growth failure, relative macrocephaly, prominent forehead, and feeding difficulties in early infancy (72-75). In contrast to SRS, limb asymmetry is an infrequent finding (73). Upd(20)mat may resemble TS14 since both have features of developmental delay, growth restriction and hypotonia (72). Advanced maternal age has been reported in association with upd(20)mat (71).

The *GNAS* locus is on chromosome 20q and may be maternally or paternally imprinted depending on the tissue site. In upd(20)mat there is loss of the paternally expressed allele of the *GNAS* locus. Deficiency in paternal *GNAS* gene products (including XLas and A/B), in combination with overexpression of maternally derived GSα resulting in hypersensitivity of Gsα-mediated hormone receptors, may account for problems with energy metabolism, feeding, and growth (71, 74-76). In contrast to upd(20)mat, paternal upd(20) leads to reduction in Gsα expression, leading to parathyroid hormone (PTH) resistance and pseudohypoparathyroidism type 1b (77, 78). Growth hormone deficiency has been reported in upd(20)mat patients (74). Hypercalcemia with low/low-normal PTH has been described in children with upd(20)mat (71), possibly due to Gsα overexpression leading to PTH-receptor hypersensitivity. Impaired inactivation of 1,25(OH)2D may also contribute to hypercalcemia as a *CYP24A1* gene mutation was reported in one individual (79). Thyrotropin (TSH) receptor hypersensitivity may also feature (71). Thus, in addition to growth monitoring, individuals with upd(20)mat may require surveillance of calcium, TSH, free thyroxine (T4), and free triiodothyronine (T3) levels.

### Maternal Uniparental Disomy of Chromosome 16

Maternal uniparental disomy of chromosome 16 (upd(16)mat) may give rise to an SRS-like phenotype and has been reported in up to 2.1% of individuals with a SRS phenotype of unknown etiology (12). The phenotype may be due to aberrant expression of the chromosome 16 imprinted genes, autosomal recessive mutations unmasked as a result of chromosome 16 isodisomy, mosaic trisomy 16, or placental insufficiency caused by trisomy 16 (12, 80). The overexpression of zinc-finger gene *ZNF597* (paternally imprinted, ie, maternally expressed) situated on 16p13.3 may account for some features seen in upd(16)mat, in particular growth failure (12, 81).

Upd(16)mat individuals may exhibit IUGR and SGA, short stature, relative macrocephaly, and a prominent forehead. Other features include pregnancy-induced hypertension, clinodactyly, prematurity, congenital heart disease, hypospadias, and—like SRS—prenatal and postnatal growth failure and low BMI (12, 80). Compared to SRS, the frequency of SGA birth is lower, while the frequency of congenital heart disease is higher (12). Spontaneous catch-up growth to normal height was observed in one individual (81). Clinicians should consider genetic testing for upd(16)mat in patients with SRS-like phenotypes of unknown etiology who are born preterm and have congenital heart disease (12).

### Multi-Locus Imprinting Disturbance

Up to 30% of SRS patients with hypomethylation of H19 TSS-DMR have multi-locus imprinting disturbance (MLID) (82). Generally, one single differential methylation region (DMR) is altered in a given imprinting disorder; however, MLID occurs in a proportion of patients, in whom DNA methylation abnormalities (most commonly loss of methylation) exist at multiple imprinted loci, ie, aberrant methylation of further imprinted loci in addition to the disease-specific ones (83, 84). Cis-acting genetic mutations are usually not seen in MLID (85). Endocrine-disrupting chemicals, parental metabolic and nutritional status, assisted reproductive techniques, and trans-acting variants influencing oocyte development (in the mother) or early epigenetic reprogramming, have been suggested as contributory factors in the pathogenesis (82-84, 86).

Phenotypic diversity exists in MLID patients, which means those who do not fulfill clinical criteria for molecular testing may remain undiagnosed. It is currently not standard practice to perform full epigenomic analysis in patients with molecular imprinting disorders; thus, cases of MLID will be undiagnosed (84). The clinical heterogeneity may be due to tissue-specific epigenotypes, and the involvement of multiple loci may influence phenotype severity (87-89). The major phenotypic traits may be related to the disease-specific loci with more severe methylation abnormality or the degree of tissue-specific mosaicism (89). In patients with SRS-MLID, growth failure and additional congenital anomalies are frequently observed (89). Additionally, *MEST* gene hypomethylation in SRS-MLID has been observed (87, 90, 91). As MLID-associated features can evolve with age, following the molecular diagnosis of an imprinting disorder investigation for MLID may be indicated to guide disease surveillance (3, 87).

An individual with a molecular diagnosis of TS14 and Beckwith-Wiedemann syndrome has been reported (84), as has an individual with a molecular diagnosis of both SRS and TS14 (92, 93). The contribution of each locus to the phenotype is unpredictable, and features of each condition may occur. The clinical diagnosis is challenging as the classical phenotype may not be evident, but making a diagnosis is important to enable tailored management considering the management principles for each condition.

## Molecular Investigation of SRS

The molecular investigation of SRS must accommodate investigation for known causes of SRS as well as conditions that mimic SRS to help guide clinical management strategies (Fig. 2). The most common underlying molecular mechanisms underlying SRS are 11p15LOM and upd(7)mat. Numerous molecular changes at chromosome 11p15 (encompassing the paternally methylated imprinted control region H19/IGF2 IG-DMR) have been associated with SRS. Molecular testing measures DNA methylation of CpG dinucleotides at the H19/IGF2 IG-DMR27. The most common diagnostic test is methylation-specific multiplex ligation-mediated PCR amplification (MS-MLPA) which also enables copy number and DNA methylation analysis (94, 95). Negative molecular diagnosis on a blood sample could be explained by incomplete/low levels of H19/IGF2 IG-DMR hypomethylation. Additionally, methylation patterns vary between different tissues and cells (leucocytes, buccal swab, skin fibroblasts) (89, 91, 96). Upd(7)mat can be identified by microsatellite analysis. However, this cannot detect imprinting defects (epimutations) and requires DNA from at least one parent, so detection by MS-MLPA is usually employed. Recent recommendations suggest that patients with growth disturbance should receive first-line testing for TS14 simultaneously with SRS, when there are nonspecific or overlapping clinical features (97).

If 11p15, chromosome 7, and TS14 testing are negative, additional molecular testing should be considered. Over 30 different pathogenic CNVs have been associated with SRS, many involving the 11p15.5 region. Although CNVs can be detected by MS-MLPA, array analysis (comparative genomic hybridization [CGH] or single nucleotide polymorphism [SNP]) is useful for detailed assessment of the size and gene content of any CNV identified (40). Arrays will also detect regions of segmental isodisomy.

Monogenic causes (mutations in *PLAG1, HMGA2, CDKN1C,* and *IGF2* genes) are rare but pose a higher recurrence risk (2, 19, 32). These genes should be included in next-generation sequencing testing approaches. In undiagnosed cases, analysis for conditions with phenotypes overlapping with SRS, such as TS14 (if not already tested as part of first-line investigations), upd(20)mat, upd(16)mat, and MLID, should be undertaken.

Whole-genome sequencing, which scrutinizes the entirety of the genome at single-base resolution can be employed as a single test. This method potentially facilitates the identification of numerous genetic aberrations, including pathogenic monogenic variants, uniparental disomies, and CNVs. Crucially, whole-genome sequencing methodologies might overlook methylation anomalies (16). Genome-wide methylation screening should also be considered where available to detect MLID, for example, EPIC array epigenome analysis which interrogates CpGs across the genome.

## Back to the Patients

### Case 1

Molecular testing did not reveal 11p15 hypomethylation or upd(7)mat. Given the atypical clinical phenotype of developmental delay and microcephaly in association with a 3/6 NH-CSS score, further molecular testing was initiated. Whole-exome sequencing excluded an *IGF1R* gene defect but identified a maternally inherited, heterozygous damaging *HMGA2* gene variant, confirming a rare monogenic cause of SRS. The identification of a genetic cause for the patient's phenotype allowed an end to diagnostic testing, the initiation of active surveillance, and referral for genetic counseling.

### Case 2

At 4.5 years, the patient developed recurrent early morning symptomatic hypoglycemia and investigations confirmed ketotic hypoglycemia. Weight gain continued and calorie restriction was required. As the clinical course was atypical for SRS, molecular investigations for conditions with phenotypes overlapping with SRS were instigated, revealing Temple syndrome (TS14) secondary to maternal uniparental disomy of chromosome 14. Diagnosis prompted active surveillance for precocious puberty and by the age of 5 years, she developed breast budding with biochemical evidence of GDPP (luteinizing hormone–releasing hormone test [U/L]: baseline LH 0.3, FSH 2.6; peak LH 33.6, FSH 17.8). GnRHa treatment was commenced, resulting in breast tissue regression and pubertal arrest.

### Summary

SRS is clinically and molecularly heterogeneous and an underlying (epi)genetic cause is currently identifiable in ∼60% cases. Stratification of the molecular subtype is critical to guide appropriate clinical management. In recent years, there have been significant developments in this area that are relevant to clinical practice. Conditions with phenotypes overlapping with SRS which “mimic” SRS and fulfill NH-CSS criteria can be diagnosed as “clinical SRS” if standard (epi)genetic investigations are negative. However, these conditions require tailored management and differing care strategies to classical SRS cases.

Detailed molecular investigations, including next-generation sequencing approaches, are key in the diagnosis of rarer monogenic causes of SRS. It is notable that the NH-CSS is poor at identifying many of these cases missing 60% *PLAG1*, 47% *CDKN1C*, 35% *HMGA2* and 14% *IGF2* cases. Additionally, the presence of associated or atypical clinical features, including microcephaly (OFC > 2 SD below the mean for age, sex, and ethnicity) and learning difficulties, should not preclude clinicians from investigating for rarer causes of SRS. This emphasizes the need to extend the molecular investigation of apparent and atypical SRS to inform clinical management decisions and enhance outcomes for affected individuals.

## Data Availability

Original data generated and analyzed during this study are included in this published article and in a data repository listed in References (44).

## Abbreviations

11p15LOM: loss of methylation of chromosome 11p15
BMI: body mass index
CDK1N1C: Cyclin-dependent kinase inhibitor 1C
CNV: copy number variant
DMR: differential methylation region
GDPP: gonadotrophin-dependent precocious puberty
GH: growth hormone
GNRHa: gonadotropin-releasing hormone analogue
HMGA2: high-mobility group AT-hook 2
IGF-1: insulin-like growth factor 1
IGF1R: insulin-like growth factor 1 receptor
IGF2: insulin-like growth factor 2
IGFBP: insulin-like growth factor binding protein
IUGR: intrauterine growth restriction
MLID: multi-locus imprinting disturbance
MS-MLPA: methylation-specific multiplex ligation-mediated PCR amplification
NH-CSS: Netchine-Harbison Clinical Scoring System
OFC: occipito-frontal circumference
PLAG1: pleomorphic adenoma gene 1
PTH: parathyroid hormone
PWS: Prader-Willi syndrome
rHGH: recombinant human growth hormone
SGA: small for gestational age
SRS: Silver-Russell syndrome
upd(7)mat: maternal uniparental disomy of chromosome 7
upd(20)mat: maternal uniparental disomy of chromosome 20
TS14: Temple syndrome

## References

[1] Walenkamp MJE , Robers JML, Wit JM, et al Phenotypic features and response to GH treatment of patients with a molecular defect of the IGF-1 receptor. J Clin Endocrinol Metab. 2019;104(8):3157‐3171. 30848790 10.1210/jc.2018-02065

[2] Wakeling EL , Brioude F, Lokulo-Sodipe O, et al Diagnosis and management of Silver–Russell syndrome: first international consensus statement. Nat Rev Endocrinol. 2017;13(2):105‐124. 27585961 10.1038/nrendo.2016.138

[3] Bakker B , Sonneveld LJH, Woltering MC, Bikker H, Kant SG. A girl with Beckwith-Wiedemann syndrome and pseudohypoparathyroidism type 1B due to multiple imprinting defects. J Clin Endocrinol Metab. 2015;100(11):3963‐3966. 26367199 10.1210/jc.2015-2260

[4] Fuke T , Mizuno S, Nagai T, et al Molecular and clinical studies in 138 Japanese patients with Silver-Russell syndrome. PLoS One. 2013;8(3):e60105. 23533668 10.1371/journal.pone.0060105 PMC3606247

[5] Wakeling EL , Amero SA, Alders M, et al Epigenotype-phenotype correlations in Silver-Russell syndrome. J Med Genet. 2010;47(11):760‐768. 20685669 10.1136/jmg.2010.079111 PMC2976034

[6] Bruce S , Hannula-Jouppi K, Peltonen J, Kere J, Lipsanen-Nyman M. Clinically distinct epigenetic subgroups in Silver-Russell syndrome: the degree of H19 hypomethylation associates with phenotype severity and genital and skeletal anomalies. J Clin Endocrinol Metab. 2009;94(2):579‐587. 19017756 10.1210/jc.2008-1805

[7] Azzi S , Salem J, Thibaud N, et al A prospective study validating a clinical scoring system and demonstrating phenotypical-genotypical correlations in Silver-Russell syndrome. J Med Genet. 2015;52(7):446‐453. 25951829 10.1136/jmedgenet-2014-102979 PMC4501172

[8] Netchine I , Rossignol S, Dufourg M-N, et al 11p15 imprinting center region 1 loss of methylation is a common and specific cause of typical Russell-Silver syndrome: clinical scoring system and epigenetic-phenotypic correlations. J Clin Endocrinol Metab. 2007;92(8):3148‐3154. 17504900 10.1210/jc.2007-0354

[9] Bliek J , Terhal P, van den Bogaard M-J, et al Hypomethylation of the H19 gene causes not only Silver-Russell syndrome (SRS) but also isolated asymmetry or an SRS-like phenotype. Am J Hum Genet. 2006;78(4):604‐614. 16532391 10.1086/502981 PMC1424698

[10] Bartholdi D , Krajewska-Walasek M, Ounap K, et al Epigenetic mutations of the imprinted IGF2-H19 domain in Silver-Russell syndrome (SRS): results from a large cohort of patients with SRS and SRS-like phenotypes. J Med Genet. 2009;46(3):192‐197. 19066168 10.1136/jmg.2008.061820

[11] Yamaguchi KT , Salem JB, Myung KS, Romero AN, Skaggs DL. Spinal deformity in Russell–Silver syndrome. Spine Deform. 2015;3(1):95‐97. 27927458 10.1016/j.jspd.2014.06.003

[12] Inoue T , Yagasaki H, Nishioka J, et al Molecular and clinical analyses of two patients with UPD(16)mat detected by screening 94 patients with Silver-Russell syndrome phenotype of unknown aetiology. J Med Genet. 2019;56(6):413‐418. 30242100 10.1136/jmedgenet-2018-105463 PMC6582712

[13] Binder G , Ziegler J, Schweizer R, et al Novel mutation points to a hot spot in CDKN1C causing Silver-Russell syndrome. Clin Epigenetics. 2020;12(1):152. 33076988 10.1186/s13148-020-00945-y PMC7574352

[14] Sabir AH , Ryan G, Mohammed Z, et al Familial Russell–Silver syndrome like phenotype in the PCNA domain of the *CDKN1C* gene, a further case. Case Rep Genet. 2019;2019:1‐8. 10.1155/2019/1398250 PMC6959155 31976094

[15] Brioude F , Oliver-Petit I, Blaise A, et al CDKN1C mutation affecting the PCNA-binding domain as a cause of familial Russell Silver syndrome. J Med Genet. 2013;50(12):823‐830. 24065356 10.1136/jmedgenet-2013-101691

[16] Alhendi ASN , Lim D, McKee S, et al Whole-genome analysis as a diagnostic tool for patients referred for diagnosis of Silver-Russell syndrome: a real-world study. J Med Genet. 2022;59(6):613‐622. 34135092 10.1136/jmedgenet-2021-107699

[17] Inoue T , Nakamura A, Iwahashi-Odano M, et al Contribution of gene mutations to Silver-Russell syndrome phenotype: multigene sequencing analysis in 92 etiology-unknown patients. Clin Epigenetics. 2020;12(1):86. 32546215 10.1186/s13148-020-00865-x PMC7298762

[18] Kerns SL , Guevara-Aguirre J, Andrew S, et al A novel variant in CDKN1C is associated with intrauterine growth restriction, short stature, and early-adulthood-onset diabetes. J Clin Endocrinol Metab. 2014;99(10):E2117‐E2122. 25057881 10.1210/jc.2014-1949 PMC4184067

[19] Abi Habib W , Brioude F, Edouard T, et al Genetic disruption of the oncogenic HMGA2-PLAG1-IGF2 pathway causes fetal growth restriction. Genet Med. 2018;20(2):250‐258. 28796236 10.1038/gim.2017.105 PMC5846811

[20] Begemann M , Zirn B, Santen G, et al Paternally inherited IGF2 mutation and growth restriction. N Engl J Med. 2015;373(4):349‐356. 26154720 10.1056/NEJMoa1415227

[21] Xia C-L , Lyu Y, Li C, et al Rare de novo IGF2 variant on the paternal allele in a patient with Silver–Russell syndrome. Front Genet. 2019;10:1161. 31803239 10.3389/fgene.2019.01161 PMC6872539

[22] Maharaj AV , Cottrell E, Thanasupawat T, et al Characterization of HMGA2 variants expands the spectrum of Silver-Russell syndrome. JCI Insight. 2024;9(6):e169425. 10.1172/jci.insight.169425 PMC11063932 38516887

[23] De Crescenzo A , Citro V, Freschi A, et al A splicing mutation of the HMGA2 gene is associated with Silver–Russell syndrome phenotype. J Hum Genet. 2015;60(6):287‐293. 25809938 10.1038/jhg.2015.29

[24] Hübner CT , Meyer R, Kenawy A, et al HMGA2 variants in Silver-Russell syndrome: homozygous and heterozygous occurrence. J Clin Endocrinol Metab. 2020;105(7):2401‐2407. 10.1210/clinem/dgaa273 32421827

[25] Plachy L , Strakova V, Elblova L, et al High prevalence of growth plate gene variants in children with familial short stature treated with GH. J Clin Endocrinol Metab. 2019;104(10):4273‐4281. 30753492 10.1210/jc.2018-02288

[26] Leszinski GS , Warncke K, Hoefele J, Wagner M. A case report and review of the literature indicate that HMGA2 should be added as a disease gene for Silver-Russell syndrome. Gene. 2018;663:110‐114. 29655892 10.1016/j.gene.2018.04.027

[27] Buysse K , Reardon W, Mehta L, et al The 12q14 microdeletion syndrome: additional patients and further evidence that HMGA2 is an important genetic determinant for human height. Eur J Med Genet. 2009;52(2–3):101‐107. 19298872 10.1016/j.ejmg.2009.03.001

[28] Vado Y , Pereda A, Llano-Rivas I, Gorria-Redondo N, Díez I, Perez de Nanclares G. Novel variant in PLAG1 in a familial case with Silver-Russell syndrome suspicion. Genes (Basel). 2020;11(12):1461. 33291420 10.3390/genes11121461 PMC7762056

[29] Meyer R , Begemann M, Hübner CT, et al One test for all: whole exome sequencing significantly improves the diagnostic yield in growth retarded patients referred for molecular testing for Silver–Russell syndrome. Orphanet J Rare Dis. 2021;16(1):42. 33482836 10.1186/s13023-021-01683-x PMC7821667

[30] Chen W , Gruber A, Kyriss M, Bend E, Sullivan R, Keppler-Noreuil K. P145: microcephaly in atypical Silver-Russell syndrome caused by defects in PLAG1. Genet Med Open. 2023;1(1):100174.

[31] Lokulo-Sodipe O , Ballard L, Child J, et al Phenotype of genetically confirmed Silver-Russell syndrome beyond childhood. J Med Genet. 2020;57(10):683‐691. 32054688 10.1136/jmedgenet-2019-106561 PMC7525777

[32] Saal HM , Harbison MD, Netchine I. Silver-Russell syndrome. In: Adam MP, Feldman J, Mirzaa GM, *et al*., eds. *GeneReviews*^®^ [Internet]. University of Washington; 2002. https://www.ncbi.nlm.nih.gov/books/NBK1324/20301499

[33] Gicquel C , Rossignol S, Cabrol S, et al Epimutation of the telomeric imprinting center region on chromosome 11p15 in Silver-Russell syndrome. Nat Genet. 2005;37(9):1003‐1007. 16086014 10.1038/ng1629

[34] Sachwitz J , Meyer R, Fekete G, et al NSD1 duplication in Silver-Russell syndrome (SRS): molecular karyotyping in patients with SRS features. Clin Genet. 2017;91(1):73‐78. 27172843 10.1111/cge.12803

[35] Inoue T , Nakamura A, Fuke T, et al Genetic heterogeneity of patients with suspected Silver-Russell syndrome: genome-wide copy number analysis in 82 patients without imprinting defects. Clin Epigenetics. 2017;9(1):52. 28515796 10.1186/s13148-017-0350-6 PMC5433143

[36] Price SM , Stanhope R, Garrett C, Preece MA, Trembath RC. The spectrum of Silver-Russell syndrome: a clinical and molecular genetic study and new diagnostic criteria. J Med Genet. 1999;36(11):837‐842. 10544228 PMC1734267

[37] Zhu M , Gong C, Wu D, Huang S, Cao B. [Analysis of clinical and genetic characteristics of 20 cases of children with Silver Russell syndrome]. Zhonghua Er Ke Za Zhi. 2013;51(3):216‐220. 23751585

[38] Binder G , Seidel A-K, Martin DD, et al The endocrine phenotype in Silver-Russell syndrome is defined by the underlying epigenetic alteration. J Clin Endocrinol Metab. 2008;93(4):1402‐1407. 18230663 10.1210/jc.2007-1897

[39] DeChiara TM , Efstratiadis A, Robertsen EJ. A growth-deficiency phenotype in heterozygous mice carrying an insulin-like growth factor II gene disrupted by targeting. Nature. 1990;345(6270):78‐80. 2330056 10.1038/345078a0

[40] Ishida M . New developments in Silver-Russell syndrome and implications for clinical practice. Epigenomics. 2016;8(4):563‐580. 27066913 10.2217/epi-2015-0010 PMC4928503

[41] Eggermann T , Begemann M, Binder G, Spengler S. Silver-Russell syndrome: genetic basis and molecular genetic testing. Orphanet J Rare Dis. 2010;5(1):19. 20573229 10.1186/1750-1172-5-19 PMC2907323

[42] Voss R , Ben-Simon E, Avital A, et al Isodisomy of chromosome 7 in a patient with cystic fibrosis: could uniparental disomy be common in humans? Am J Hum Genet. 1989;45(3):373‐380. 2570528 PMC1683410

[43] Hensen K , Braem C, Declercq J, et al Targeted disruption of the murine *Plag1* proto-oncogene causes growth retardation and reduced fertility. Dev Growth Differ. 2004;46(5):459‐470. 15606491 10.1111/j.1440-169x.2004.00762.x

[44] Kurup U , Lim D, Palau H , et al Supplemental data: approach to the patient with suspected Silver-Russell syndrome. Mendeley Data, V1, doi: 10.17632/8dfn4m8z29.1 https://data.mendeley.com/datasets/8dfn4m8z29/1 PMC11403326 38888172

[45] Li J , Chen L-N, He H-L. CDKN1C gene mutation causing familial Silver-Russell syndrome: a case report and review of literature. World J Clin Cases. 2023;11(19):4655‐4663. 37469742 10.12998/wjcc.v11.i19.4655 PMC10353515

[46] Eggermann K , Bliek J, Brioude F, et al EMQN best practice guidelines for the molecular genetic testing and reporting of chromosome 11p15 imprinting disorders: Silver-Russell and Beckwith-Wiedemann syndrome. Eur J Hum Genet. 2016;24(10):1377‐1387. 27165005 10.1038/ejhg.2016.45 PMC5027690

[47] Masunaga Y , Inoue T, Yamoto K, et al IGF2 mutations. J Clin Endocrinol Metab. 2020;105(1):116‐125. 10.1210/clinem/dgz034 31544945

[48] Liu D , Wang Y, Yang X-A, Liu D. De novo mutation of paternal IGF2 gene causing Silver–Russell syndrome in a sporadic patient. Front Genet. 2017;8:105. 28848601 10.3389/fgene.2017.00105 PMC5550680

[49] Yamoto K , Saitsu H, Nakagawa N, et al De novo IGF2 mutation on the paternal allele in a patient with Silver–Russell syndrome and ectrodactyly. Hum Mutat. 2017;38(8):953‐958. 28489339 10.1002/humu.23253

[50] Poulton C , Azmanov D, Atkinson V, et al Silver Russel syndrome in an aboriginal patient from Australia. Am J Med Genet A. 2018;176(12):2561‐2563. 30152198 10.1002/ajmg.a.40502

[51] Rockstroh D , Pfäffle H, Le Duc D, et al A new p.(Ile66Serfs*93) IGF2 variant is associated with pre- and postnatal growth retardation. Eur J Endocrinol. 2019;180(1):K1‐K13. 30400067 10.1530/EJE-18-0601

[52] Loid P , Lipsanen-Nyman M, Ala-Mello S, et al Case report: a novel de novo IGF2 missense variant in a Finnish patient with Silver-Russell syndrome. Front Pediatr. 2022;10:969881. 36268036 10.3389/fped.2022.969881 PMC9578642

[53] Binder G , Eggermann T, Weber K, Ferrand N, Schweizer R. The diagnostic value of IGF-2 and the IGF/IGFBP-3 system in Silver-Russell syndrome. Horm Res Paediatr. 2017;88(3–4):201‐207. 28675902 10.1159/000477666

[54] Demars J , Gicquel C. Epigenetic and genetic disturbance of the imprinted 11p15 region in Beckwith-Wiedemann and Silver-Russell syndromes. Clin Genet. 2012;81(4):350‐361. 22150955 10.1111/j.1399-0004.2011.01822.x

[55] Begemann M , Spengler S, Gogiel M, et al Clinical significance of copy number variations in the 11p15.5 imprinting control regions: new cases and review of the literature. J Med Genet. 2012;49(9):547‐553. 22844132 10.1136/jmedgenet-2012-100967 PMC3439641

[56] Hokken-Koelega ACS , van der Steen M, Boguszewski MCS, et al International consensus guideline on small for gestational age: etiology and management from infancy to early adulthood. Endocr Rev. 2023;44(3):539‐565. 36635911 10.1210/endrev/bnad002 PMC10166266

[57] Storr HL , Chatterjee S, Metherell LA, et al Nonclassical GH insensitivity: characterization of mild abnormalities of GH action. Endocr Rev. 2019;40(2):476‐505. 30265312 10.1210/er.2018-00146 PMC6607971

[58] Ioannides Y , Lokulo-Sodipe K, Mackay DJG, Davies JH, Temple IK. Temple syndrome: improving the recognition of an underdiagnosed chromosome 14 imprinting disorder: an analysis of 51 published cases. J Med Genet. 2014;51(8):495‐501. 24891339 10.1136/jmedgenet-2014-102396

[59] Kagami M , Nagasaki K, Kosaki R, et al Temple syndrome: comprehensive molecular and clinical findings in 32 Japanese patients. Genet Med. 2017;19(12):1356‐1366. 28640239 10.1038/gim.2017.53 PMC5729347

[60] Geoffron S , Abi Habib W, Chantot-Bastaraud S, et al Chromosome 14q32.2 imprinted region disruption as an alternative molecular diagnosis of Silver-Russell syndrome. J Clin Endocrinol Metab. 2018;103(7):2436‐2446. 29659920 10.1210/jc.2017-02152

[61] Juriaans AF , Kerkhof GF, Mahabier EF, et al Temple syndrome: clinical findings, body composition and cognition in 15 patients. J Clin Med. 2022;11(21):6289. 36362517 10.3390/jcm11216289 PMC9656486

[62] Hordijk R , Wierenga H, Scheffer H, Leegte B, Hofstra RM, Stolte-Dijkstra I. Maternal uniparental disomy for chromosome 14 in a boy with a normal karyotype. J Med Genet. 1999;36(10):782‐785. 10528860 10.1136/jmg.36.10.782 PMC1734247

[63] Binder G , Schweizer R, Blumenstock G, Ferrand N. Adrenarche in Silver-Russell syndrome: timing and consequences. J Clin Endocrinol Metab. 2017;102(11):4100‐4108. 28945864 10.1210/jc.2017-00874

[64] Dauber A , Cunha-Silva M, Macedo DB, et al Paternally inherited DLK1 deletion associated with familial central precocious puberty. J Clin Endocrinol Metab. 2017;102(5):1557‐1567. 28324015 10.1210/jc.2016-3677 PMC5443333

[65] Sánchez-Solana B , Nueda ML, Ruvira MD, et al The EGF-like proteins DLK1 and DLK2 function as inhibitory non-canonical ligands of NOTCH1 receptor that modulate each other's activities. Biochim Biophys Acta. 2011;1813(6):1153‐1164. 21419176 10.1016/j.bbamcr.2011.03.004

[66] Yuan G , Zhang X, Liu S, Chen T. Chinese familial central precocious puberty with hyperuricemia due to recurrent DLK1 mutation: case report and review of the literature. Mol Genet Genomic Med. 2022;10(12):e2087. 36353763 10.1002/mgg3.2087 PMC9747546

[67] Brightman DS , Lokulo-Sodipe O, Searle BA, et al Growth hormone improves short-term growth in patients with temple syndrome. Horm Res Paediatr. 2018;90(6):407‐413. 30836360 10.1159/000496700

[68] Stalman SE , Kamp GA, Hendriks YMC, Hennekam RCM, Rotteveel J. Positive effect of growth hormone treatment in maternal uniparental disomy chromosome 14. Clin Endocrinol (Oxf). 2015;83(5):671‐676. 26119964 10.1111/cen.12841

[69] Severi G , Bernardini L, Briuglia S, et al New patients with Temple syndrome caused by 14q32 deletion: genotype-phenotype correlations and risk of thyroid cancer. Am J Med Genet A. 2016;170A(1):162‐169. 26333654 10.1002/ajmg.a.37346

[70] Benetatos L , Hatzimichael E, Londin E, et al The microRNAs within the DLK1-DIO3 genomic region: involvement in disease pathogenesis. Cell Mol Life Sci. 2013;70(5):795‐814. 22825660 10.1007/s00018-012-1080-8 PMC11114045

[71] Kawashima S , Nakamura A, Inoue T, et al Maternal uniparental disomy for chromosome 20: physical and endocrinological characteristics of five patients. J Clin Endocrinol Metab. 2018;103(6):2083‐2088. 29878129 10.1210/jc.2017-02780

[72] Hjortshøj TD , Sørensen AR, Yusibova M, et al upd(20)mat is a rare cause of the Silver-Russell-syndrome-like phenotype: two unrelated cases and screening of large cohorts. Clin Genet. 2020;97(6):902‐907. 32087029 10.1111/cge.13727

[73] Mulchandani S , Bhoj EJ, Luo M, et al Maternal uniparental disomy of chromosome 20: a novel imprinting disorder of growth failure. Genet Med. 2016;18(4):309‐315. 26248010 10.1038/gim.2015.103

[74] Tannorella P , Minervino D, Guzzetti S, et al Maternal uniparental disomy of chromosome 20 (UPD(20)mat) as differential diagnosis of Silver Russell syndrome: identification of three new cases. Genes (Basel). 2021;12(4):588. 33920573 10.3390/genes12040588 PMC8073552

[75] Yu S , Gavrilova O, Chen H, et al Paternal versus maternal transmission of a stimulatory G-protein alpha subunit knockout produces opposite effects on energy metabolism. J Clin Invest. 2000;105(5):615‐623. 10712433 10.1172/JCI8437 PMC289181

[76] Plagge A , Gordon E, Dean W, et al The imprinted signaling protein XL alpha s is required for postnatal adaptation to feeding. Nat Genet. 2004;36(8):818‐826. 15273686 10.1038/ng1397

[77] Hanna P , Grybek V, Perez de Nanclares G, et al Genetic and epigenetic defects at the GNAS locus lead to distinct patterns of skeletal growth but similar early-onset obesity. J Bone Miner Res. 2018;33(8):1480‐1488. 29693731 10.1002/jbmr.3450 PMC6105438

[78] Bastepe M , Jüppner H. GNAS locus and pseudohypoparathyroidism. Horm Res. 2005;63(2):65‐74. 15711092 10.1159/000083895

[79] Hureaux M , Chantot-Bastaraud S, Cassinari K, et al When a maternal heterozygous mutation of the CYP24A1 gene leads to infantile hypercalcemia through a maternal uniparental disomy of chromosome 20. Mol Cytogenet. 2021;14(1):23. 33952337 10.1186/s13039-021-00543-4 PMC8101107

[80] Scheuvens R , Begemann M, Soellner L, et al Maternal uniparental disomy of chromosome 16 [upd(16)mat]: clinical features are rather caused by (hidden) trisomy 16 mosaicism than by upd(16)mat itself. Clin Genet. 2017;92(1):45‐51. 28032339 10.1111/cge.12958

[81] Yamazawa K , Inoue T, Sakemi Y, et al Loss of imprinting of the human-specific imprinted gene ZNF597 causes prenatal growth retardation and dysmorphic features: implications for phenotypic overlap with Silver-Russell syndrome. J Med Genet. 2021;58(6):427‐432. 32576657 10.1136/jmedgenet-2020-107019 PMC8142457

[82] Begemann M , Rezwan FI, Beygo J, et al Maternal variants in NLRP and other maternal effect proteins are associated with multilocus imprinting disturbance in offspring. J Med Genet. 2018;55(7):497‐504. 29574422 10.1136/jmedgenet-2017-105190 PMC6047157

[83] Bilo L , Ochoa E, Lee S, et al Molecular characterisation of 36 multilocus imprinting disturbance (MLID) patients: a comprehensive approach. Clin Epigenetics. 2023;15(1):35. 36859312 10.1186/s13148-023-01453-5 PMC9979536

[84] Grosvenor SE , Davies JH, Lever M, Sillibourne J, Mackay DJG, Temple IK. A patient with multilocus imprinting disturbance involving hypomethylation at 11p15 and 14q32, and phenotypic features of Beckwith-Wiedemann and Temple syndromes. Am J Med Genet A. 2022;188(6):1896‐1903. 35266280 10.1002/ajmg.a.62717 PMC9310769

[85] Elbracht M , Mackay D, Begemann M, Kagan KO, Eggermann T. Disturbed genomic imprinting and its relevance for human reproduction: causes and clinical consequences. Hum Reprod Update. 2020;26(2):197‐213. 32068234 10.1093/humupd/dmz045

[86] Sanchez-Delgado M , Riccio A, Eggermann T, et al Causes and consequences of multi-locus imprinting disturbances in humans. Trends Genet. 2016;32(7):444‐455. 27235113 10.1016/j.tig.2016.05.001

[87] Fontana L , Bedeschi MF, Maitz S, et al Characterization of multi-locus imprinting disturbances and underlying genetic defects in patients with chromosome 11p15.5 related imprinting disorders. Epigenetics. 2018;13(9):897‐909. 30221575 10.1080/15592294.2018.1514230 PMC6284780

[88] Poole RL , Docherty LE, Al Sayegh A, et al Targeted methylation testing of a patient cohort broadens the epigenetic and clinical description of imprinting disorders. Am J Med Genet A. 2013;161A(9):2174‐2182. 23913548 10.1002/ajmg.a.36049

[89] Azzi S , Blaise A, Steunou V, et al Complex tissue-specific epigenotypes in Russell-Silver syndrome associated with 11p15 ICR1 hypomethylation. Hum Mutat. 2014;35(10):1211‐1220. 25044976 10.1002/humu.22623

[90] Azzi S , Rossignol S, Steunou V, et al Multilocus methylation analysis in a large cohort of 11p15-related foetal growth disorders (Russell Silver and Beckwith Wiedemann syndromes) reveals simultaneous loss of methylation at paternal and maternal imprinted loci. Hum Mol Genet. 2009;18(24):4724‐4733. 19755383 10.1093/hmg/ddp435

[91] Begemann M , Spengler S, Kanber D, et al Silver-Russell patients showing a broad range of ICR1 and ICR2 hypomethylation in different tissues. Clin Genet. 2011;80(1):83‐88. 20738330 10.1111/j.1399-0004.2010.01514.x

[92] Abi Habib W , Brioude F, Azzi S, et al Transcriptional profiling at the DLK1/MEG3 domain explains clinical overlap between imprinting disorders. Sci Adv. 2019;5(2):eaau9425. 30801013 10.1126/sciadv.aau9425 PMC6382400

[93] Brück J , Begemann M, Dey D, Elbracht M, Eggermann T. Molecular characterization of temple syndrome families with 14q32 epimutations. Eur J Med Genet. 2020;63(12):104077. 33010492 10.1016/j.ejmg.2020.104077

[94] Eggermann T , Schönherr N, Eggermann K, et al Use of multiplex ligation-dependent probe amplification increases the detection rate for 11p15 epigenetic alterations in Silver-Russell syndrome. Clin Genet. 2008;73(1):79‐84. 18070127 10.1111/j.1399-0004.2007.00930.x

[95] Scott RH , Douglas J, Baskcomb L, et al Methylation-specific multiplex ligation-dependent probe amplification (MS-MLPA) robustly detects and distinguishes 11p15 abnormalities associated with overgrowth and growth retardation. J Med Genet. 2008;45(2):106‐113. 18245390 10.1136/jmg.2007.053207

[96] Russo S , Calzari L, Mussa A, et al A multi-method approach to the molecular diagnosis of overt and borderline 11p15.5 defects underlying Silver-Russell and Beckwith-Wiedemann syndromes. Clin Epigenetics. 2016;8:23. 26933465 10.1186/s13148-016-0183-8 PMC4772365

[97] Eggermann T , Monk D, de Nanclares GP, et al Imprinting disorders. Nat Rev Dis Primers. 2023;9(1):33. 37386011 10.1038/s41572-023-00443-4

